# Building Community Resilience via Developing Community Capital toward Sustainability: Experiences from a Hakka Settlement in Taiwan

**DOI:** 10.3390/ijerph18179012

**Published:** 2021-08-26

**Authors:** Rung-Jiun Chou, Feng-Tzu Huang

**Affiliations:** 1 Department of Landscape Architecture, Chung Yuan Christian University, Taoyuan City 32023, Taiwan; rungjiun@cycu.edu.tw; 2 Liberal Arts Center/Department of Nursing, Da-Yeh University, Changhua 51591, Taiwan

**Keywords:** resilience, community capital, food landscape, community care, health, well-being

## Abstract

Developing community capital is widely viewed as a way to address community resilience-related issues toward sustainability. Based on a Taiwanese, peri-urban, Hakka settlement, this article presents findings on the practical factors in the development of community capital via farm-to-table and community care, and their implications for a resilient, healthy community. It shows that community capital arising from the pond farming, pond education, and community service systems can interact to support its diversity and linkability. The pond-based social network is identified as the key to mitigating the impacts of community challenges regarding food safety concerns, environmental degradation, and aging population. It argues that the pond-based food landscape, communal network, and a borderless campus can enhance community capital as well as play significant parts in achieving community sustainability by promoting residents’ health and well-being.

## 1. Introduction

Community, as a term possessing numerous definitions and usages in different subjects, has been a significant and interesting discipline in social science, yet sometimes it is hard to explain exactly what a community is [1]. Based on a general idea of a community as “people in interaction and a geographic area… where interaction occurs and where such interaction is considered community living” ([2] p. 118), communities could be viewed as the entirety of the interactions of social networks in a clear geographic space [3], such as a territorially arranged population with a network of relations, belonging, and identity [4]. The domain of a community may be a politically defined civic district, an economically defined urban zone, an environmentally defined rural village, or a socially defined neighborhood, and the basis of community ties could be diverse involving family, ethnicity, proximity, social class, or religion [1]. However, it is argued that the meaning of communities involving such territorial limitations and social bonds may not be necessary in contemporary society [5] where social media are promoting new ideas of communities with identities involving people dispersed throughout the world. In short, there are many different types of communities covering many different kinds of human organizations or relationships. Therefore, this study will largely confine itself to the discussion of community development for geographically bounded communities, i.e., communities of place rather than communities of interest.

While the use of the term “community” could mainly include two notions—territorial and relational [6]—with both being equally important and not mutually exclusive [7], this study maintains the focus on community as a crucial field for addressing a range of development issues. A number of studies suggest that the idea of resilience has been developed in response to community development [8,9,10], with various forms of community capital, such as human capital, social capital, and other resources and infrastructure [11]. For example, a community’s social capital is viewed as a key factor in the community’s vulnerability and resilience to environmental changes and uncertainties such as flooding [12]. In short, as the notion of resilience in the community structure is applicable to the sustainable future of a community faced with challenges from the environment and society, a shift of emphasis from an ultimate target of sustainability to a continuing action of establishing resilience is meaningful. Therefore, it is necessary to examine the community as a whole, the interrelationships of the diverse resources within a community, and how such resources collectively develop community resilience aiming for community sustainability. Therefore, the research asks: what factors are related to community resilience for the pond-based community? This qualitative study focuses on a peri-urban Hakka settlement in Taiwan to explore the building of community health and well-being via the development of community capital. The significance of the case study is to better-inform policy and practice while promoting community development in the East Asian region. Subsequently, this paper will review the interrelations of community capital, resilience, health, and well-being, and will explain the study site and methods of data collection and analysis, followed by an exploration of the process of developing various forms of capital at the community level. The important issues about creating community capital in response to a pond-based social network toward community resilience, health and well-being will then be discussed and conclusions presented.

## 2. Community Capital, Resilience, Health, and Well-Being

Stemming from the Latin word—*resilio*—meaning “to bounce back” [13], resilience has evolved various concepts and usages in dissimilar intellectual traditions, such as engineering, psychology, disaster risk management, and socio-ecological systems which make a long-term contribution to the resilience-related literature [14]. According to Folke [15], Klein et al. [13], Meerow and Newell [16], and Meerow et al. [17], modern resilience research originated from Holling [18] arguing that an ecosystem with resilience is able to resist disturbances, bounce-back, and rebuild itself when needful. While it is related to human-social systems, as Dohwe and Kwangwama [19] indicate, resilience allows humans to anticipate and respond to changes and events in the uncertain future. Early work mainly dealt with individuals (e.g., children or adults with psychosocial variables), yet recent studies have been concerned with more inclusive systems (e.g., general or particular kinds of groups and communities) [20,21]. Regardless of the origin of resilience, there is evidence to show that it can be developed and promoted to strengthen a positive process of adaption despite adversity. Duchek [22] conceptualizes resilience as a capability and a process with three successive resilience stages (anticipation, coping, and adaptation). She identifies the main capabilities assigned to each of these stages. Given that resilience has various meanings in the context of integrated systems of people and nature, it might be argued that moving beyond the similarities and differences of definitions and stressing that this study, as it seeks to explore community capital as a source of development, views resilience as the ability of a community to manage and recover in an effective and efficient way from the impacts of adversity. This relates to Bento and Couto’s [23] contribution to the understanding of community resilience by identifying contextual factors and the geographic, psychological, and ecological aspects that are involved, and that resilience is also about learning and developing new structures as unexpected events take place instead of only maintaining existing structures. Additionally, as shown by The World Bank [24], the strength of community resilience is mainly reliant on the achievement of retaining the community’s essential basic structures and services.

As the evolution of a community is relevant to the notion of resilience, it is suggested that there are two approaches to think about the role of community in the community-resilience interrelation: “community as context” where the community as the local environment supplies the factors (driving or restraining) affecting its resilience, and “community as an agent of change” where the focus is on the extent to which the community itself displays resilience [11]. To put it simply, in which ways and to what extent can communities take action to respond to challenges and foster resilience toward sustainability? This indicates the importance of the process of establishing community strengths that encourage resilient conditions, and this process relies heavily on identifying the primary components and mechanisms through which community strengths can be built and act to shape a collective capacity of facing adversity and forming resilience [11,25,26]. Furthermore, how the community operates as a collective unit is dependent on diverse forms of community capital as the primary components [27] and on active local organizations with neighborhood networks as the primary mechanisms [28]. In brief, community capacity works through the interactions of community capital and organizational resources within a given community whereby the conditions supporting resilience can be improved.

The notion of community is often based on a shared sense of place, which primarily involves the establishment of community resources or assets, i.e., community capital, broadly grouped into seven categories: natural, cultural, human, social, political, financial, and built [27]. The interrelationship of these categories displays a dynamic process in which all the categories are interacting with each other and achieving community resilience and making a long-term contribution toward community health and well-being. There have been many discussions on the community capital framework, such as Flora [29] about categories of community capital fostering a community ecosystem, economic security, and social inclusion; Hancock [30] about the role of community capital in the development of healthy communities; Zhu [31] about social capital, such as communal space, to support local attachment and public participation; Tong et al. [32] about the development of migrant communities where social relationships are vital; Sørensen [33] about rural communities whose development is largely reliant upon bonding social capital, such as trust, participation, and reciprocity; and Wang et al. [34] about the mutual dependency between community capital and economic activities. More importantly, the concept of community capital can shape an integrated approach to analyze the factors in community resilience. As Mayunga [35], Wilson [36], and Cocklin and Alston [37] indicate, the significance of community capital is in connecting resilience with ecological, environmental, health-linked, economic, sociological, and other aspects of discussions, theoretically within a pyramid-shaped framework displaying how the lower-level community capital categories serve as the foundation for the higher-level ones [38].

Regarding Taiwan, Lin et al. [39] indicate that communities are prone to the dilemma of autonomy in the process of establishing resilience. On the one hand, disadvantaged communities need external support, but are prone to over-reliance on government funding or professional guidance, which can be detrimental to independence without external support. On the other hand, the grassroots may be desperate for autonomy and resist external assistance, which causes problems for the government or non-profit organizations which feel, and even create, a conflict of power. In response to this ‘giving and receiving’ issue, Djalante et al. [40] suggest maintaining a flexible leadership strategy, whereby well-informed leaders and local people trust each other thereby providing the community with incentives for people’s engagement and communication. This is a favorable condition for empowering communities to develop resilience. Additionally, the development of community resilience affects the robustness of local governance, which is critical to equity and justice in the development of local communities, so it is important that community residents develop the resilience to cope with adversity. However, the existing literature in Taiwan has paid insufficient attention to the process of building community resilience, and thus more research is needed.

Ostrom [41] argues for the multi-faceted nature of human behavior in interacting with ecosystems. In the governance of “common-pool resources”, it is possible to develop a long-term co-existence with ecosystems through the successful “self-governance” mechanisms of people who use the shared resources. Norris et al. [42] suggest that continuous interaction, collaboration, and supportive relationships between community members lead to dense social networks which together play a key role in effective support services for adversity-affected people and organizations. Mathbor [43] indicates that effective use of social capital, such as social networks, social cohesion, social interaction, and solidarity helps to build the resilient communities essential for disaster prevention and management, and which can enhance the sustainability of individuals, groups, communities, and organizations. Islam and Walkerden [44] and Zhao [45] show that the connection between community members helps them to obtain emotional and spiritual support, which promotes resilience. The bonds between community members and the social support for life are vital to the community’s development in adversity [46]. Moreover, the integration of disaster prevention and community development, the formation of collaborative relationships between individuals and community groups, and the introduction of professional teams into the community, can bring diverse civil forces into grassroots communities for handling development issues [39], such as food safety, environmental degradation, and population change. The notion of collaborative disaster prevention needs to be further implemented in grassroots communities and transformed into local disaster prevention knowledge [47,48], and endogenous disaster prevention and response strategies [42]. However, local knowledge may also form a barrier to community disaster preparedness. The ingrained perceptions, a tendency to a form of ‘group-think’, and the political considerations and wait-and-see attitudes of local people could prevent innovative approaches to community disaster preparedness.

Following Western et al. [49], showing a diverse picture of community development practices, this case study of Hakka settlements in Taiwan aims to explore how community capital, which derives from the pond farming system, the pond education system, and the community service system, has formed a pond-based social network that ultimately contributes to the resilient development of a disadvantaged community faced with adversities such as food safety concerns, environmental pollution, and an aging population. The recent socio-environmental transformations in Taiwan and the background of Gaoyuan will now be briefly explained.

## 3. Background of the Study Area

Geographically, Taiwan is located in the Pacific Rim seismic zone and on the path of the Western Pacific typhoons, making it a place where earthquakes, floods, landslides, and mudslides have been frequent since ancient times. As Grano [50] indicates, Taiwan, with its rapid industrialization, urbanization, and economic growth in recent decades, has experienced many environmental problems. While this circumstance has improved greatly since the 1990s, several issues remain difficult. Certainly, some of these are global concerns such as greenhouse gas emissions and acid rain, while others are connected with local problems, which are caused by both development choices and by geographical and natural characteristics. Human impact on the natural environment is noticeable by damage to the local ecology as well as to cultivated land.

With environmental concerns, food safety is gradually considered an attribute of trust, rather than an attribute of search or experience [51]. In modern society, with increasing differentiation and division of labor, social exchanges occur over long physical and social distances. Consumers must delegate responsibilities to chains of strangers representing various organizations to complete their food purchasing tasks [52]. While consumers know less about what they are eating, it is with such a fundamental feeling of food safety that more impersonal relations of trust can be established. Community-supported agriculture (CSA), as a typical example of bringing local farmers and consumers together to build cooperation and trust, has become increasingly popular in Taiwan [53]. Although CSA has not yet been demonstrated in Gaoyuan, it is currently important for promoting the production of non-toxic vegetables through community agriculture [54].

In Taiwan, a low birth rate and an aging population are nationwide demographic problems, mainly due to a falling marriage rate, or people waiting longer to get married, and a declining birthrate coupled with medical advances, increased living standards, and longer life spans [55,56]. The government needs to formulate a suitable approach to cope with the extensive socio-economic impact of the demographic imbalance on the country. As Taiwan has become an aged society, the Ministry of Health and Welfare has promoted the creation of a community-based, long-term, care service system to support local ageing persons and enable them to enjoy their later years in a familiar environment. However, these promotional activities are inadequate [57]. Lee et al. [58] stress that with a declining birthrate, an ageing population, high housing prices, low wages, and financial difficulties in the provision of social insurance, Taiwanese society is faced with growing challenges such as a weakening ability for family care-giving, insufficient government tax revenues, and a lack of stable financial resources for developing a sound long-term care system for an ageing society in the face of limited physical resources and increasing demands. In an international context, the relationship between an ageing population and community resilience is an increasingly important issue, such as Cohen et al. [59] suggesting that older people in good health may contribute positively to building community resilience in response to a crisis, and Aldrich and Kyota [60] emphasizing the connections of the elderly residents to society so as to build community resilience for future crises.

**Figure 1:**
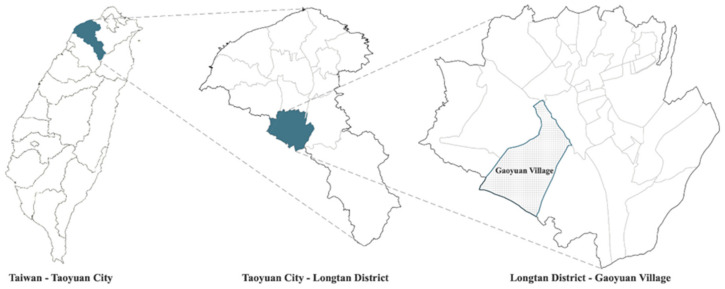
Location of Gaoyuan Village.

The study was conducted at Gaoyuan Village, a peri-urban Hakka settlement located in Longtan District, Taoyuan City (Figure 1), which is situated in the northwest of Taiwan and one of the six special municipalities. Gaoyuan is a rural community, formerly known as “Tong Luo Circle”, and is one of the highest areas on the Taoyuan Plateau which dominates the city’s landscape with its relatively flat topography. The soil is mostly infertile red clay and the community is sparsely populated. As there is no major river for irrigation, the lack of water resources led to ponds being built across the region in the early days for crop irrigation which reached its peak in the 1950s. These ponds have been a prominent feature in the landscape, and such agricultural facilities are normally situated in the comparatively flat lowland areas where it is appropriate to store rainwater and collect surface runoff. Nevertheless, industrialization and economic development have reduced the number of ponds. This has led to a gradual decline in the irrigation function of the pond system. Many ponds were buried because of land demands, or abandoned due to agricultural decline. Numbers have declined dramatically with many of the associated plants and animals now on the verge of extinction, making large changes to the local environment. It is therefore imperative to protect the original ecological functions of the ponds. The unpredictable heavy rainfall usually damages agricultural roads and leads to crop losses, as well as posing a significant threat to the lives and properties of residents.

It is noteworthy that the Hakka, who originally migrated from Mainland China to Taiwan, are descended from Han Chinese people. It is because the migration process was faced with numerous difficulties and adverse environments that their culture tends to be more diligent, conservative, and unpretentious [61]. Gaoyuan had a long history of tea and brick making owing to the region’s particular geology, geomorphology and weather conditions. However, industrial transition has led to a downturn in the local businesses and population emigration. In order to revitalize the abandoned land, the community has developed a part of a disused tea plantation into organic farms for vegetable cultivation, forming a mutually beneficial mechanism between the farm owners and the community. Additionally, in the 2010s, the community protested against the construction of a gas-fired power plant in the area of Gaoyuan. With a high level of environmental awareness and civic participation, the community finally rejected the government policy. The community has been cautious of government measures which seem to treat the village as a site for unwelcome facilities and possible environmental pollution [62].

## 4. Methods

According to Yin [63], thinking about a single case as the unique case for research, the significance of Gaoyuan is as follows. First, the village is a representative Hakka settlement with traditional-agricultural-village-like qualities, located in a peri-urban area. In the face of adversities involving a low birth rate, population aging, and environmental threats, as explained earlier, this real-life instance shows how community capital could be redeveloped by local people as an ideal state wherein village revitalization could be fulfilled more comprehensively. Second, Gaoyuan recently aims to become an environmental education site attracting many visits from groups and organizations, resulting from its developing repute as an example of pond-based community agriculture. Third, the Gaoyuan Community Development Association (GCDA), a local proactive voluntary organization, has fostered long-term community health and well-being. GCDA comprises local residents and is normally divided into the farming group, the kitchen group, the community care group, and the environmental protection group. Furthermore, as Taiwan has become an aging society, the Hakka settlement is no exception. While the working-age population lives with the elderly and goes out to work during the daytime on working days, caring for the elderly has become an issue that must be solved. Thus, GCDA aims to develop a community care service system to address the above problem, whereby old people can enjoy their later years in a warm atmosphere.

### Data Collection

Overall, the study was carried out from August 2018 to July 2020, with data coming from interviews, fieldwork, and participant observation, whereby its data source triangulation was ensured to strengthen the trustworthiness and rigor of the research findings. First, interviews were of two types: in-depth interviewing and group interviewing. Purposive sampling was used to make sure that the sample contained a variety of individual backgrounds with first-hand, long-term involvement in local development affairs. Finally, a total of 17 participants individually joined in in-depth interviews (Table 1), and 2 group interviews were conducted publicly in the community center to discuss the progress of community development.

Three main questions were asked in interviews (see below) without a specific order, aiming to prompt follow-up questions and ensure the talks responded to the research problem. The interview questions were formulated on the basis of the literature review regarding community resilience [11,25] and community capital [27,30], and preliminary fieldwork. Interviews were digitally recorded for later transcription, line-by-line coding, and analysis.

1. What are the capital resources of Gaoyuan Village that have influenced local development?
2. What is your experience in local community affairs?
3. What are the challenges and opportunities as Gaoyuan Village develops in the direction of community health and well-being?

Fieldwork was integrated with participant observation in the study to include watching, natural conversations, field notes, drawings, and photographs, with the aim of understanding the natural setting and people’s experiences, values, beliefs, and way of life, by observing and participating in the village’s activities within the ordinary life of Gaoyuan. The research team, including eight members in total, got involved in the routine and special activities of the village, such as companionship for children and the elderly, community agriculture (e.g., vegetables, fruits, and mugwort), community meals, educational courses, environmental maintenance, and facility construction (e.g., artificial floating islands), in an attempt to compare and contrast the interview data. Totally the research team conducted nine periods of participant observation adopting the role of volunteers during a period of three months, with 122 person-sessions providing 56 h of observation (Table 2). Overall, triangulation is not just about validation of data from multiple sources, but about mapping out and understanding more fully the richness and complexity of the Hakka settlement. While the collected data were divergent, further data collection measures were conducted, including fieldwork and participant observation, in an attempt to resolve any discrepancies.

Regarding ethical considerations, with dual roles as a researcher and observer, a critical self-reflection so as not to influence the field studied and data collected is important. Each of the participants was assigned a specific ID code to maintain anonymity and confidentiality in the investigation process. Moreover, the study did not recruit the elderly and children as interview participants because they were all vulnerable groups by virtue of social status and being too old and too young respectively to express their opinions fully. Concerning research limitations, the data collection activities were carried out during the daytime on normal working days, therefore the interview participants were largely from the main figures involved in community affairs rather than the working-age population who regularly go out to work. If more ordinary people’s views were included during the data-collection stage, the themes that emerged could be further elaborated from a different perspective.

**Figure 2:**
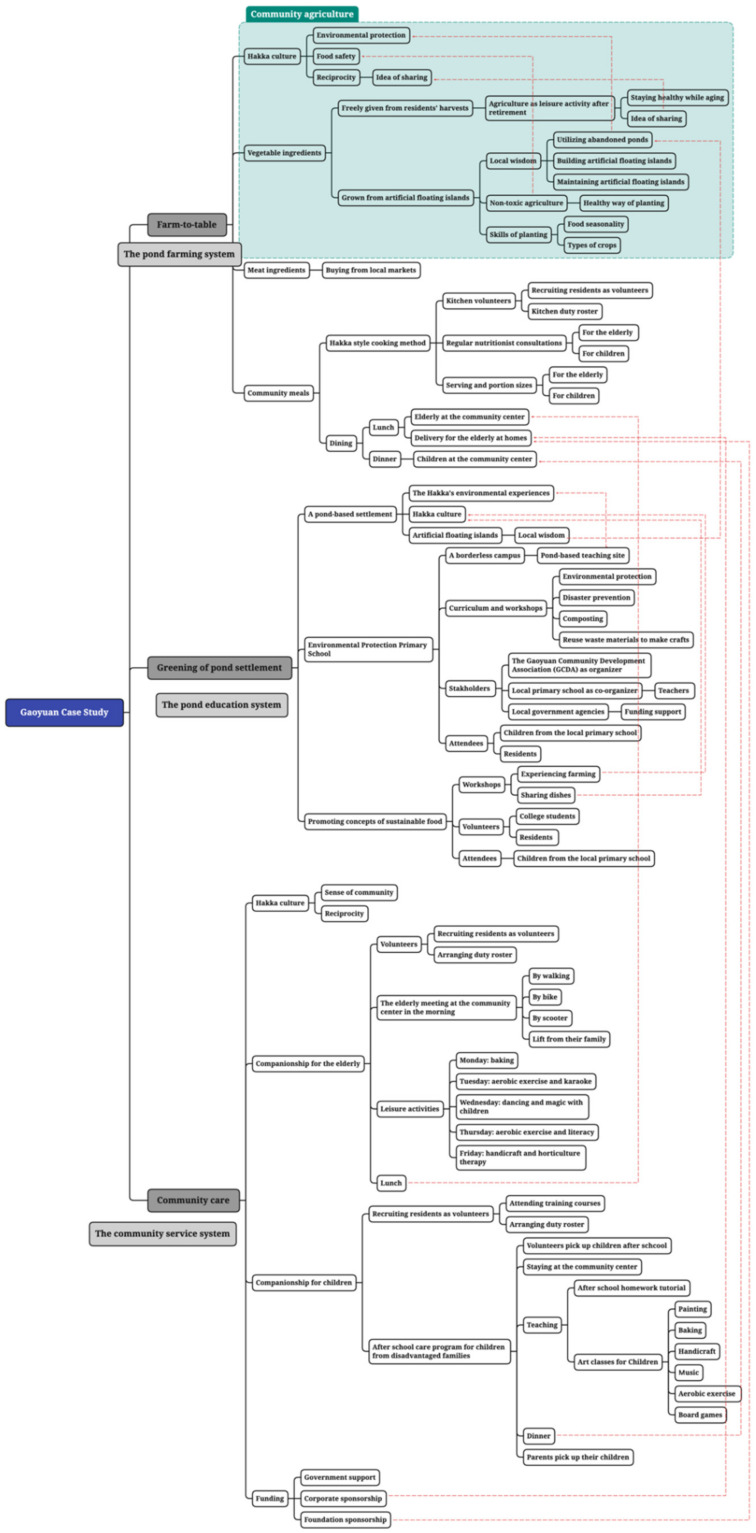
Thematic mapping of interview analysis.

In terms of data analysis, thematic analysis, defined as “a method for identifying, analyzing and reporting patterns (themes) within data” ([64] p. 79), is a flexible and useful qualitative approach to analysis [65] and was thus employed in the qualitative study, with the intention of providing a rich and detailed account of themes within the data. A number of interesting findings are revealed from the process, such as community branding, the cultural landscape, and rural tourism. However, this article will focus on three themes closely related to the study topic: (1) the pond farming system, (2) the pond education system, and (3) the community service system. The thematic mapping of interview analysis is presented in Figure 2.

## 5. Results

### 5.1. The Pond Farming System

The place where people were born and grew up is likely to be one of their most familiar environments. In general, people have a certain sense of identity with this place which is often the starting point for their participation in public affairs. Interviewee IV13, a community resident, said:

*“The main reason for participating in community-building is that I want to make the rural environment, my hometown, a better place to live, so that young people are willing to come back to live … they are willing to stay. With people moving in, then the community will be more vigorous.”*

People’s happiness comes from the welfare of, and belonging to, the community, and their satisfaction comes from the care of volunteers. Interviewee IV14, a community resident, said:

*“Every week I walk a long way to come to the community center, yet I am happy because of the concern from the volunteers. I feel that I am still valued by someone, instead of saying that I am useless when I am old.”*

Human touch is a crucial factor in fostering positive relationships among community residents. Interviewee IV15, a community environmental counselor, said:

*“The volunteers here are very kind to those who come to help them. For example, I just helped them a little last time, they immediately took out a bunch of vegetables they grew and let me take them home. Such a human touch cannot be measured by money.”*

**Figure 3:**
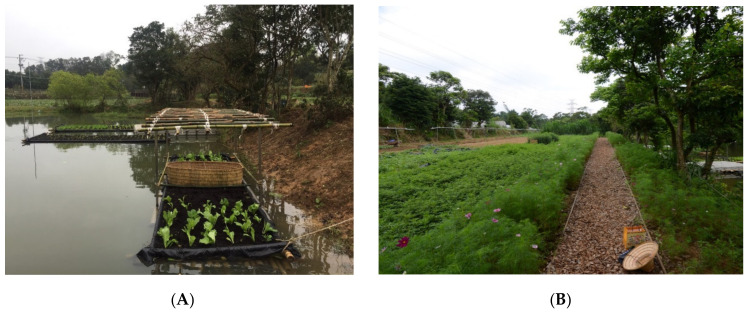
(**A**) Artificial floating islands located in ponds; (**B**) food farms situated in vacant lands.

Led by GCDA, farm-to-table is a newly-emerging type of local movement which has played a vital role in stimulating the development of community capital in Gaoyuan, faced with challenges such as food safety and environmental pollution. The community agriculture-related activities are displayed in diverse forms underpinned by locally-specific resources, such as non-toxic agriculture on both artificial floating islands located in ponds, and food farms situated in vacant lands (Figure 3), to supply benefits regarding cultivation, learning, sharing, care, food traceability, and environmental management. It indicates that farm-to-table, a typologically varied drive for greater local development, plays multiple roles in the building of community resilience. Interviewee IV1, the village chief, said:

*“It [farm-to-table] is an important local system in promoting a strong connection of people, places, ponds, and environment. We have used the vacant, state-owned lands and abandoned ponds to widely grow edible plants for community meal services.”*

Especially the pond-based farming shows an adaptive method of establishing a unique community strength that supports sufficient availability and accessibility of vegetables in the community meal services, wherein food safety, food freshness, and food seasonality can be steadily maintained. According to the village secretary, interviewee IV2, the vegetables are mainly pumpkin, melon, gourd, cucumber, and beans in summer, and mainly lettuce, cabbage, spinach, and radish in winter. The well-grown greens constitute the essential ingredients for community meal services.

By participant observation, it is found that green vegetables are grown by the farming group, and meals (lunches and dinners) are prepared from Monday to Friday by the kitchen group. The members of both groups are elderly residents, who range mostly in age from 70 to 90. Respectively, the elderly and children are the target participants of the morning and afternoon programs run in the community center. Combined with the scheduled activities, roughly 50 lunch meals and 25 dinner meals are served per day. More importantly, GCDA appoints a nutritionist to advise the kitchen group on what to eat and how to cook in order to lead a healthier lifestyle. Interviewee IV2, the village secretary, said:

*“The lunch and dinner menu is regularly examined by a general hospital’s nutritionist to satisfy the needs of the elderly and children. We will know and improve if there is a lack of essential nutrients.”*

The kitchen task is noteworthy for its organization and coordination. In cooperation with the farming group, the kitchen group is further divided into five teams, and each of them is responsible for one day of duties required in the kitchen, such as ingredient preparation, cooking, washing, and cleaning. Since the start of the services, the day-to-day operations of the five teams are fixed and therefore the members clearly realize the details of the kitchen tasks and their coordination with the scheduled activities. Interviewee IV1, the village chief, said:

*“Normally, a meal contains four dishes and one soup, served with rice or noodles, and dessert. The main dish is fish, meat or sausage, with one side dish of tofu, egg or other stir-fry recipes, and two side dishes of vegetables … So each team knows the serving size of its duty day. For example, the courses that we have on Monday and about how many people will attend and have lunch and dinner respectively.”*

According to participants’ accounts, apart from non-toxic vegetables from both artificial floating islands and food farms, they still need to buy other ingredients and groceries from markets, with a generous monthly budget of NT 40,000 supported by the local government. Despite the fact that farm-to-table in Gaoyuan cannot source all the dishes locally and so only some ingredients are labelled as local, the trend becomes apparent with a growing awareness of a bottom-up approach to building community capital. As a local government official, who is interviewee IV12, said:

*“Unlike a top-down way of community development that often shows a standardized process and result, the progress in Gaoyuan is unique in being established via local human strengths and environmental resources. I think this is the key difference.”*

This account of local development characteristics corresponds with interviewee IV11, a team leader of the community care group, who emphasized the importance of the Hakka people’s spirit and culture of farming that contributes to the dedicated practice of farm-to-table. Using artificial floating islands as an example, the account of interviewee IV9, who is a farmer and also an environmental education teacher, indicates that the process of conducting such a water planting system is learning-by-doing. He said:

*“We have tried different ways of farming, on land and water, which are different. For example, we once failed in spinach growing on water … trial and error … we learned from it, indeed.”*

This is echoed by interviewees IV7 and IV8, the team leaders of the farming group, on the implementation process of artificial floating islands, wherein seasons of farming, types of vegetables, characteristics of vegetables, construction of facilities (Figure 3A), and upkeep of amenities are fundamental for the fulfilment of farm-to-table and the implications for food safety. Regarding environmental benefits, the pond-based farming reduces pollution by using bamboos, instead of plastic materials, as the main structure of an artificial floating island, and by reducing the use of pesticides due to plants being grown on water. Additionally, the pond-based farming takes advantage of abandoned ponds, and there is no need for the practice to have further watering. This helps mitigate environmental depletion and increases ecological diversity, such as artificial floating islands becoming part of the habitat for frogs.

**Figure 4:**
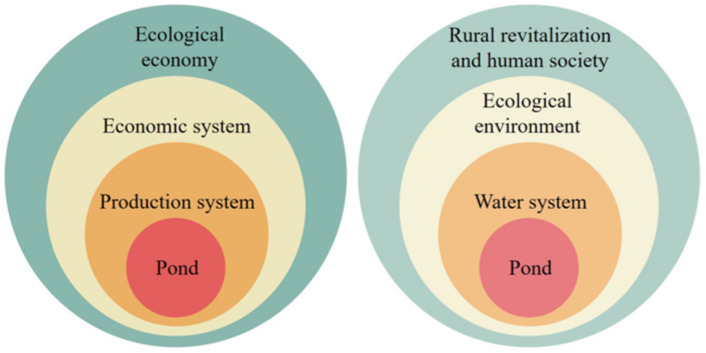
Two development approaches for the pond-based community.

Under the leadership of GCDA, the pond farming system has enhanced the local sense of community. Participant observation suggests that Gaoyuan is undergoing pond-based development reliant on two specific approaches (Figure 4). One sees the pond as the basic production system which lies at the heart of the community’s economic system aimed at the goal of an ecological economy. Another has the pond as the center of the local water system with a major role in maintaining the ecological environment and contributing to the diversity of rural revitalization and human society.

By leveraging community resources, the pond farming system has become a key feature of Gaoyuan, where ponds, artificial floating islands, farming, and meal services are closely interconnected with culture, life, and the environment. The local small-scale farming integrated with the practice of farm-to-table fosters a solid foundation for an active, cohesive social network. This is essential within a changing society where in the uncertain future the process of developing a variety of capital categories at the community level is seen as beneficial to the desired end-state of a resilient, healthy community.

### 5.2. The Pond Education System

Through participant observation, the research team members participated in the community’s environmental protection education. It has established the community-level “Environmental Protection Primary School”, which incorporates multiple stakeholders such as local primary school teachers, NGOs, local government agencies, and attendees like children and residents. Through the curriculum and workshops, the concepts of environmental protection and disaster prevention are explored. The attendees also practice composting and the reuse of waste materials to make environmentally friendly crafts, which helps to build a consensus supporting environmental protection via practical work. The curriculum is implemented in the field rather than in the classroom, with the idea of a borderless campus, where the pond-based environment is the center of the teaching site. Interviewee IV16, a school teacher, said:

*“Gaoyuan is a typical Hakka settlement and is also one of the demonstration sites of the Environmental Protection Agency … the pond in the community is a good field for environmental education. For example, some of the aquatic plants are unique to the region and we are trying to restore them.”*

Another salient feature of environmental education in Gaoyuan is called sustainable food with the two themes of “land” and “food”. The recruited voluntary college students and voluntary community residents are invited to work together to promote the concept of sustainable food. By participant observation, it is found that sustainable food comprises two parts: experiencing farming, and sharing dishes. Through workshops, experiencing farming involves the improvement of the farming environment, and the wise use of land for production, so that children can appreciate the nourishment of the land and learn to value food without wasting it. Sharing dishes allows children to experience self-sufficiency and hands-on cooking, and learn to appreciate and cherish food resources by sharing.

Through the pond education system, the Hakka’s environmental experiences and local wisdom are linked together, showing the spirits of hard work and reciprocity typical of the Hakka culture. The pond education system is the focus of environmental protection and disaster prevention activities thereby strengthening the community’s resilience. Through the GCDA network, the related information is further disseminated, and participants can work together to address potential disasters and environmental problems. This approach is beneficial to shaping a localized and autonomous resilient community in its original context. Gaoyuan is being developed as a pond-based settlement. The pond education system serves as a bridge between the residents and the community, enabling people to know the surroundings and showing a practical combination of environmental protection and disaster prevention so as to build local community resilience.

### 5.3. The Community Service System

Community care is essentially general, non-medical, care services to assist in personal-related daily activities. In Gaoyuan, the community care group of GCDA is responsible for the day-to-day operations of care, supported by the farm-to-table system. Interviewee IV1, the village chief, emphasized that they have all been working toward a common goal of a heart-warming village, particularly giving special importance to the care of the elderly and children. A team leader of the community care group, interviewee IV11, values the provision of community care services very highly as a key to unite the community. She said:

*“As a community care center, we have done it well, not for some people, but for the whole community. From old age to child, they are all being taken care of, such as the offering of community activities that intend to serve each level of people. This helps the cohesion of the people, the identity of the community.”*

The main part of community care services in Gaoyuan includes meal services, and companionship for children and the elderly. Firstly, the provision of meals is based on the farm-to-table system explained in Section 5.1. According to interviewee IV10, a team leader of the environmental protection group, the year 2016 was significant for the introduction of the artificial floating island method which has gradually become a well-established steady practice that links the pond-based food landscape with community services. Furthermore, the lunch meal services for the elderly (over 65 years old), and the dinner meal services for children, in cooperation with the community life schedule, have been provided free of charge since 2006 and 2015 respectively. Additionally, the free meal services incorporate home-delivered meals. According to the village secretary, interviewee IV2, the program of home-delivered meals has been financially aided by a local high-tech company—AU Optronics Corporation—for more than ten years, and currently there are two community volunteers delivering daily lunch and dinner meals to 27 old people who live alone and are unable to purchase or prepare their own food. This indicates the multiple benefits of integrating the service of home-delivered meals with the visits to the old people who require more attention.

Secondly, it is found by participant observation that companionship for the elderly is achieved via a range of special activities held in the community center, which include baking, aerobic exercise, karaoke, dancing, magic (with children), literacy, handicraft, and horticulture therapy. According to the statistics, there are approximately 500 residents over 65 years old, and about 100 of them (once up to 120) regularly attend the related activities. It is also found that their ways of reaching the community center mainly involve walking, riding a bike or scooter, or lifts from their family. Nevertheless, it is important to note that the extent of community care services is limited to within a roughly 1.5 km radius of the community center, owing to a lack of convenient public transportation. In other words, the attendance at the activities, to a large extent, is restricted to those old people who can drive their own vehicles or can arrive at the center by foot. In general, the attendance at the specially arranged programs is viewed by all interviewees as a key to promoting physical and mental health of the elderly. As the village chief, Interviewee IV1, said:

*“Here becomes the elderly’s domain of daily life … we always call them to come here, otherwise they are just watching TV at home all day, from channel 1 to channel 100. We always stress that coming here means that you earn about NT$30,000 a month, as you come here regularly and thus you don’t need to hire a personal caregiver.”*

Correspondingly, interviewee IV11, who is a team leader of the community care group, mentioned that her father greatly appreciates the companionship services for the elderly that supply him with a comfortable environment for aging in place. Moreover, interviewee IV7, a team leader of the farming group, mentioned that the long-term presence in the elderly companionship-related programs can enrich their lives, and naturally they can enjoy better social networking. Indeed, it shows that the community center has become the core of healthy living for the elderly.

Thirdly, the child companionship-related services of community care in Gaoyuan have been financially supported by the CTBC Charity Foundation’s project entitled ‘Love For Kids’ since 2015, with the aim of offering diverse companionships for disadvantaged children, through combining local and external resources. By participant observation, it is found that this has been fulfilled through providing nutritious meals on the basis of the farm-to-table system, and a range of special courses such as painting, baking, handicraft, music, aerobic exercise, language, and board games, in addition to homework tutorials. Different from the general after-school tutoring class supplied in the elementary school, these care services are highly reliant on community volunteers (i.e., GCDA) to build a community-wide social safety network for children.

The community center is a suitable place for implementing the child companionship-related activities. Interviewee IV3, a coordinator of companionship affairs for children, pointed out that this is clearly not a profitable daycare business, but a non-profit amenity where children can grow and learn. According to the statistics, there are now 18 children, ranging from first grade to sixth grade, who regularly attend the programs. Nevertheless, the community care group encountered a number of difficulties in the implementation process. For example, a lack of parental support, and a gap between planning and execution raised by interviewee IV3; parent-teacher communication, and classroom management indicated by interviewee IV4; teaching methods as revealed by interviewee IV5; and the long-term participation and coordination of the group members mentioned by interviewee IV6.

As the child companionship-related services develop and expand, the group members need to attend the childcare-associated educational classes regularly, such as parent education, child psychology, and communication skills, which can help them deal on-site with various situations, difficulties, and challenges. Clearly, this is crucial in making progress toward the building of community resilience aiming for community sustainability. Such importance is underpinned by the account of interviewee IV3, a coordinator of child companionship affairs, who revealed more details about the implementation process:

*“Actually, grandparenting would be a fundamental issue in the childcare. Parents are busy with work and have limited time to take care of their children. We even know children’s personality, abilities, and learning in school better than their parents, and therefore we usually advise parents on how to deal with children’s problems. Also, how we communicate with a macho father and his wife and child, is a common situation. This is a great issue we have faced in communication with parents.”*

The service of accompanying children, which mixes community and external resources, is really a consequential category of community capital for disadvantaged families and children. Interviewee IV6, who is a team leader of the community care group, emphasized that children’s education deeply influences their future learning, growth, and adaptation to society. Interviewee IV4, an art teacher with years of experience of teaching disadvantaged children, shows that it is necessary to pay special attention to their concentration, emotional management, and learning attitude. Additionally, the processes and procedures must be handled carefully because of their identity considerations. On the other hand, in the process of caring for disadvantaged children, the group members must also improve themselves by the necessary educational classes explained earlier. As interviewee IV6 said:

*“Emotion regulation and relationship management, such as how to get along with, teach, and lead these children, have always been quite challenging for me.”*

**Figure 5:**
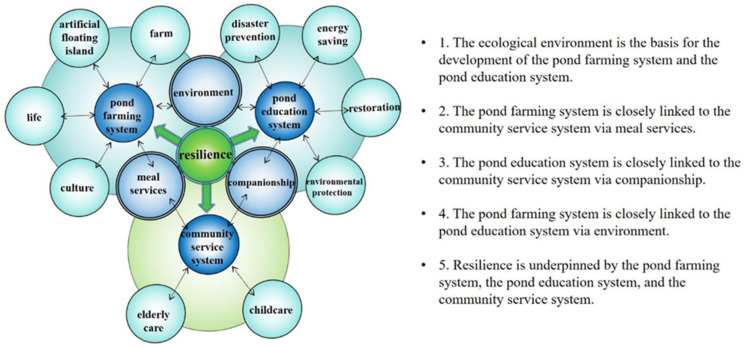
The dynamic, interrelated network of Gaoyuan as a pond-based community seeking to develop resilience.

Overall, as shown in Figure 5, the pond farming system produces non-toxic vegetables and provides healthy meal services, which are integrated with the care of the elderly and children, allowing the community service system in Gaoyuan to achieve highly consistent results. The pond education system implements community-oriented environmental education, using the pond as a demonstration site that integrates disaster prevention with environmental-protection-related activities. With different types of community capital embedded in an interrelated network, the Hakka settlement is situated in the front line of societal development confronting a low birth rate, population aging, demographic changes, climate change, and environmental degradation by trying to demonstrate what a resilient community may look like.

## 6. Development of Community Capital in the Pond-Based Hakka Settlement

The case study of Gaoyuan shows that the nature of a community involves groups of people who interact with one another and fulfil their needs on the basis of the social systems and organizations. These individuals live in a place and share a sense of identity, wherein a relationship among people, culture, and the environment (natural and built) related to a specific area exists. This is generally supported by the literature reviewed [2,3,4,5,6,7] stressing the entirety of social networks and interactions in a clear, meaningful, geographic space. Therefore, community development pays special attention to what local people do in order to improve the quality of such relationships in a locality. While groups of people in a geographically defined area can interact for mutual support, the ways of their interaction form the structures and customs of the locality, and such structures and customs also construct their activities and daily life. The development experience of Gaoyuan has consolidated the arguments of the community resilience-related studies mentioned earlier, from more general ideas [8,9,10,11,12] to specific cases, such as Norris et al. [42] about social networks for adversity-affected people and organizations; Mathbor [43] about social cohesion for disaster prevention and management; Islam and Walkerden [44] and Zhao [45] about social connections for emotional and spiritual support; and Brown and Schafft [46] about social bonds for community development in transition.

In line with Chaskin [11], Berkes and Ross [25], and Kulig et al. [26], the case study of the Hakka settlement shows the development of a variety of community capital categories as an effective approach for building the capacity of a community facing the impacts of adversity. Especially the pond-based food landscape acts as a catalyst for the integrated implementation of community services. This further empirically shows a Taiwanese example of Chaskin’s [11] argument about community as context as well as an agent of change, and of Breton’s [28] argument about physical organizations and non-physical networks as the primary mechanisms, in the light of community resilience. Echoing Cocklin and Alston [37], the research findings indicate that community capital is far more than simply the attributes of a community. Environmentally speaking, the effects of community capital on the neighborhood landscape, such as quality and diversity, are noticeable. Socially speaking, the categories of community capital, such as the pond-based farming, meal services, and companionship for children and the elderly, exhibit the robustness of local citizenship that fosters community solidarity, cohesive networking, and local revitalization. Ecologically speaking, community capital works as part of the regional ecological system, such as artificial floating islands, to the benefit of aquatic ecosystems, green lifestyles, and wildlife habitats. Economically speaking, the actual practice of community capital, such as community care based on the mix of local and external resources, aims to fulfil the role of social services without considering economic profitability.

In detail, community capital in Gaoyuan can be further classified into several categories, none of which can prevail in isolation. This community development practice in Taiwan, unlike Flora et al. [27], further expands as well as demonstrates an understanding of community resilience in the East Asian region, via a Hakka settlement. Natural capital contains the area’s water, soil, weather, landscape, vegetation, and wildlife, wherein the flat topography, lateritic soils, humid climate, pond-scape, pond-side vegetation, and pond-based wildlife feature in the peri-urban environment of the Hakka settlement. This natural capital not only constitutes the basic elements of the environment, but also represents both opportunities and challenges for the resilient development of Gaoyuan. For example, as a type of community agriculture, non-toxic agriculture on artificial floating islands is seen as an environmentally sound practice, as a result of a wise local response to the limits of geomorphological conditions, abandoned water resources, the regional weather pattern, and the advantage of pond-related scenery, flora, and fauna. It shows that natural capital affects people’s actions and functions as the basis on which all the other categories of capital rely, echoing Flora [29] about community ecosystems as the main community capital fostering economic security and social inclusion. In Gaoyuan, the pond environment demonstrates its capacity as an ecological resource. Because of the wetland characteristics, rich habitat conditions, and good migration paths, the ponds have become the habitat for some endangered aquatic organisms in Taiwan, retained their native species and are providing a place for migratory birds to live, forage, and multiply.

Cultural capital is Hakka’s spirit and the culture of farming that shapes the group’s world view, such as how they see the world and what is valued by them. Corresponding to the brief history of Gaoyuan reviewed in Section 3, the results imply that the Hakka people are a very united ethnic group with firm ideas and actions. This has developed as a driving force to promote community affairs, such as artificial floating islands, the pond farming system, meal services, and companionship for children and the elderly. These are deeply rooted in the locality and preserve the cultural values in the changing times. It shows the inheritance of Hakka culture in the development process of community resilience that highlights the wider positive benefits for local revitalization, welfare, and the well-being of residents. More importantly, the notion of kindness is being realized via the altruistic practice of farm-to-table and community care which bring advantages to both the people and the environment. The ponds in Gaoyuan form a pond-based social network, which can link residents and the community in multiple ways.

Human capital involves the capacities and possibilities of individuals committed to local affairs. In Gaoyuan, the main human capital is the long-term hard work and dedication of the volunteers operating the community activities. They can manage work independently and collaboratively based on their skills, talents, knowledge, experience, and health. This meaning is linked with Hancock [30] highlighting health as a key component of human capital, whereby a healthy community is built, in general, with high levels of social, ecological, human, and economic capital. Additionally, the creation of community cohesion and the willingness of residents to participate in public affairs without salary are particularly valuable. It is not simply the management of the pond environment, but the pond is symbolically and practically regarded as a core place, where participants connect with each other owing to their similar interests and form an interpersonal network. For example, interviewee IV5, an art teacher, described his attitude and mentality of volunteering as that of “a happy fool” and “being a volunteer is to be selfless”; interviewee IV3, a coordinator of child companionship affairs, stressed “patience, enlightenment, and guidance” as the capabilities needed for a community care project manager.

As indicated in Aldrich [47], social capital is built on mutual relationships, wherein bonding is the extent to which an individual is linked with the community. Behind the results of the pond farming system, the pond education system, and the community service system, there is a strong person-to-place bond that develops via emotional connections, meanings, and experiences of the pond, such as childhood memories of rural life, and feelings for the land and ancestors. Such place attachment has encouraged local people to actively engage in community affairs, and the honor of participation and the sense of being valued and of accomplishment ultimately feed back into their long-term contributions. Echoing Zhu [31] who highlights the importance of community place-based social capital, such as communal space, to support neighborhood attachment and grassroots participation, the study reveals that the more nature with green common spaces a community possesses, the more possibilities the community fosters for social settings and social bonding among residents. In line with Tong et al. [32] that social networks, social norms, and trust among neighbors are crucial factors in the migrant community development, and with Sørensen [33] about bonding social capital, such as trust, rate of participation in civic associations, and measures of reciprocity that are especially beneficial to the development of rural communities, this study indicates that social capital in the Hakka settlement involves a collective identity and a sense of a shared future, embodied in the notion of “the community is your second home”, which, via the pond-based social network, supports the desired-end state: the aged are cared for until death, and children are nourished, educated, and nurtured. To put it simply, people feel more at ease in the setting and atmosphere where they feel most at home.

Built capital refers to human-made infrastructure, with the benefit of contributing to the development of other categories of community capital. In addition to roads, bridges, electricity, gasoline, the internet, gas, water systems, drainage, sewers, telecommunications, and other public utilities and facilities, which have been identified as common categories of built capital by Flora et al. [27], the findings show that the pond and the community center, as the focus of built capital, are necessities of daily life for the elderly and children in Gaoyuan. What the Hakka community is doing to be sure that local people could develop a healthier life, is to promote the community center-based services with the pond-based food landscape, in terms of the built capital that helps to make that possible.

Through this case study, it is clear that economic capital involves the role of the pond in Gaoyuan as the foundation of the community’s production system. This is related to Wang et al. [34] who suggest that a shift in rural household economic activities in Southeast China caused changes in land use and ecological restoration, where environmental deterioration occurred during the early period of economic growth, but positive impacts in rural ecosystems have become more apparent. Correspondingly, this interdependence between natural capital and human economic activities is stressed, as the pond environment is the main part of the economic system in the community.

**Figure 6:**
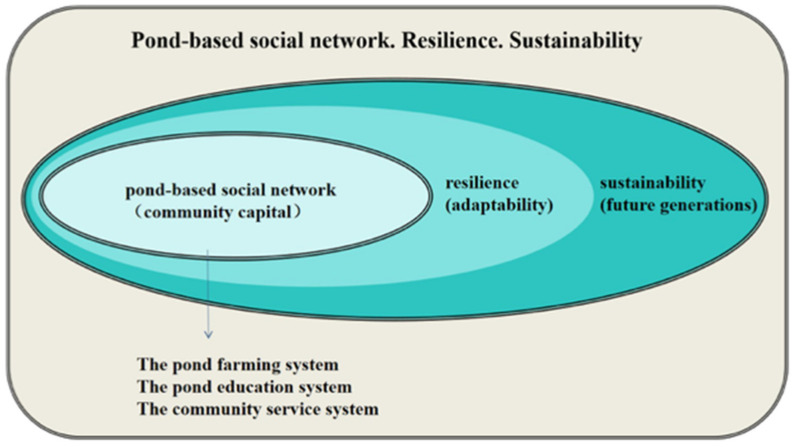
Relationship of community resilience with the pond-based social network.

Overall, as shown in Figure 5 and Figure 6, the “pond-based social network” in Gaoyuan is reliant upon the three main systems of pond farming, pond education, and community service, which display a strong, cohesive link between agriculture, artificial floating islands, meal services, companionship for children and the elderly, environmental protection, disaster prevention, the Hakka, and other factors, which are recognized in this study. Further, the development of Gaoyuan demonstrates the multifaceted values of pond resources in a rural area, such as ecological systems, land use, water and soil conservation, an edible landscape, and social cohesion. From a cultural perspective, the pond systems in Taoyuan are a potential world heritage site. From the perspective of education, they popularize the idea of environmental protection to residents, whereby environmental education should not be limited to the classroom, and the message from the real environment is the most direct and useful for the community. From an ecological point of view, ponds are viewed as a part of the regional ecosystem. From the perspective of climate change, ponds can undertake the task of disaster prevention. From a leisure point of view, ponds provide an extensive network of blue and green spaces. In line with Mayunga [35], Wilson [36], and Cocklin and Alston [37], this study generally confirms the potential of an interconnected relationship of community capital that underpins the pond-based social network.

Figure 6 illustrates that Gaoyuan’s community capital, which exists in the dynamic, interrelated systems (i.e., Figure 5, the pond farming, pond education, and community service systems), is integrated to shape a pond-based social network, thereby building community resilience through greater adaptability to different challenges and moving towards sustainability for the benefit of future generations. Specifically speaking, the use of artificial floating islands, as the key to the pond farming system, is local human wisdom to adapt existing resources and skills to new systems and operating conditions, with the benefit of dealing with the effects of community challenges such as food security and environmental pollution. Additionally, community-oriented environmental education, as in the implementation of the pond education system, provides a borderless campus to enable participants to organize themselves to increase their capacity for learning from past experiences for better environmental protection and risk reduction measures on site. Furthermore, the general, non-medical care services especially for children and the elderly, as the main part of the community service system, are an effective resident support mechanism to anticipate, prepare for, respond to, and recover from the influences of community difficulties such as a low birth rate, population aging, and demographic changes. Evidently, the arguments such as Duchek [22] indicating anticipation, coping, and adaptation as the capability and process of resilience; Bento and Couto [23] stressing the adaptation processes of resilience in complex systems, through which an interaction of experience learned and emergent knowledge is distinguished; Cohen et al. [59] showing active older people are beneficial to the development of community resilience in a crisis; and Aldrich and Kyota [60] showing the extent to which the connections of the elderly to society affect the establishment of community resilience for unexpected events or changes, support the literature reviewed previously.

Overall, the development experience relates to a range of research examining the relationship between community capital, resilience, health, and well-being. As mentioned earlier, Chaskin [11] indicates the importance of the categories of social capital as the principal components that shape contexts and local institutions as the principal mechanisms by which they could contribute to community resilience. This is expanded by the study so that the diverse community capital categories (i.e., principal components) and the vitality of GCDA (i.e., principal mechanisms) in Gaoyuan are practically fundamental to its resilient conditions toward community health and well-being. Moreover, the reviewed literature of Callaghan and Colton [38] suggests a theoretical, pyramid-shaped framework of community capital showing how the lower-level categories function as the foundation for the higher-level categories. Nevertheless, this study argues that the relations of all these categories may not be in a static hierarchy, but in a dynamic, overlapping state without a fixed hierarchical relationship. For instance, while natural and built capital perform as the physical characteristics of the environment, cultural and social capital serve as the non-physical strengths of the settlement, and all of them share in the profound effects of assisting in the establishment of other categories of community capital. Additionally, the reviewed literature of Western et al. [49] show a diverse picture of the studied communities, in which social capital is key to measuring community strength. This is echoed by the study in that a higher-level development of social capital seems to be an indicator of a stable, mature community. The experiences from Gaoyuan provide us with a sense of how a Hakka settlement evolves toward resilience through the establishment of community capital, and with a multifaceted understanding of the close connections between physical environments, culture-place-people relationships, and community services.

## 7. Conclusions

Evolving from the definition of resources and characteristics possessed by communities, the various forms of community capital have become a globally important topic as they are seen as a vehicle to address community resilience-related issues toward sustainability. By a case study of the Hakka community of Gaoyuan in Taiwan, this paper presents findings on the practical factors in the development of community capital categories and their implications for a resilient, healthy community. The main findings are:

1. The pond farming system, the pond education system, and the community service system exhibit the synergistic operation of the categories of community capital, showing the diversity and linkability of natural, cultural, human, social, built, and economic capital.
2. The pond farming system is a typologically varied drive for local development, including non-toxic agriculture, such as the use of artificial floating islands. This involves a key environmental factor—the pond-based food landscape, with the benefit of handling the impacts of community challenges such as food security and environmental pollution.
3. The pond education system, as a community-oriented environmental education project, is run with programs enabling participants to experience hands-on activities that aim to secure environmental protection and disaster prevention. This involves a key educational factor—a borderless campus—with the benefits of disseminating the related information and knowledge on site.
4. The community service system, as the general, non-medical care services to aid personal-related daily activities, covers free meal services and companionship for children and the elderly. This involves a key societal factor—the pond-based communal network, with the benefit of addressing the influences of community difficulties such as a low birth rate, population aging, and demographic changes.

The findings indicate that the resilience of the Hakka settlement is underpinned by the pond farming system, the pond education system, and the community service system, which consist of different categories of community capital and are interconnected as a pond-based social network, linking residents and the community in multiple ways. Promoting community resilience toward community health and well-being through developing categories of community capital is obviously an important topic for environmental, economic, social, and other reasons. Such community development is context-reliant and differs from place to place. Thus, a Hakka settlement could be an appropriate research context for better exploring the specific characteristics and resources that link to each other, and how they could evolve to create a more resilient environment. Further research is recommended to explore the actors participating in the complex, dynamic systems of local communities, such as driving and restraining forces for community resilience in a disadvantaged community.

The case study intends to investigate a different system of community capital categories existing in an East Asian region and to communicate the potential of community resilience from a Taiwanese perspective. Indeed, under the impact of an infectious disease such as COVID-19, Goal 3 of the SDGs—good health and well-being—cannot be achieved without building resilient societies. This is underpinned by the peri-urban case of Gaoyuan that works against the standardization and homogenization of practice, showing that the pond-based food landscape, communal network, and a borderless campus can enhance community capital and act as effective components of community health and well-being.

## Figures and Tables

**Table 1:** Demographic data of the in-depth interview participants.

ID Code	Age Group	Role	Participation	Occupation
IV1	50–59	The leader of village affairs	Over 20 years	Chief of village
IV2	50–59	A coordinator of village affairs and a team leader of the kitchen group	15–20 years	Secretary of village
IV3	50–59	A coordinator of child companion affairs	5–10 years	Project manager
IV4	Over 60	An art teacher	10–15 years	Retired
IV5	Over 60	An art teacher	5–10 years	Retired
IV6	Over 60	A team leader of the community care group	5–10 years	Retired
IV7	Over 60	A team leader of the farming group	5–10 years	Retired
IV8	Over 60	A team leader of the farming group	5–10 years	Retired
IV9	50–59	An environmental education teacher and a farmer	10–15 years	Farm owner
IV10	Over 60	A team leader of the environmental protection group	10–15 years	Retired
IV11	Over 60	A team leader of the community care group	10–15 years	Retired
IV12	50–59	A decision-maker for local policy	15–20 years	Government official
IV13	50–59	Ordinary villager	15–20 years	Community resident
IV14	Over 60	Ordinary villager	15–20 years	Community resident
IV15	40–49	External expert	5–10 years	Community environmental counselor
IV16	30–39	General volunteer	5–10 years	School teacher
IV17	30–39	General volunteer	5–10 years	School teacher

**Table 2:** Details of participant observation.

Visit	Agricultural Activities in the Pond Area	Leisure Activities for the Elderly	After-School Tutoring for Children	Meal Services in the Kitchen
1	8 members/1 h	8 members/1 h	8 members/1 h	--
2	3 members/1 h	3 members/2 h	3 members/4 h	3 members/1 h
3	5 members/1 h	5 members/2 h	5 members/4 h	5 members/1 h
4	--	4 members/3 h	4 members/3 h	4 members/1 h
5	--	4 members/3 h	4 members/3 h	4 members/1 h
6	2 members/1 h	2 members/2 h	2 members/3 h	2 members/1 h
7	--	--	6 members/3 h	6 members/1 h
8	3 members/1 h	3 members/2 h	--	3 members/1 h
9	2 members/1 h	2 members/3 h	7 members/3 h	2 members/1 h
Subtotal	23 person times 6 h	31 person times 18 h	39 person times 24 h	29 person times 8 h
Total	122 person-sessions providing 56 h of participant observation			

## Data Availability

Not applicable.
